# Furosemide-Induced Thrombotic Thrombocytopenic Purpura: A Report of a Rare Case

**DOI:** 10.7759/cureus.25689

**Published:** 2022-06-06

**Authors:** Taruna Chandok, Zaheer A Qureshi, Laura Yapor, Misbahuddin Khaja

**Affiliations:** 1 Internal Medicine, BronxCare Health System, Bronx, USA; 2 Internal Medicine, Icahn School of Medicine at Mount Sinai, New York, USA; 3 Pulmonary and Critical Care, BronxCare Health System, Bronx, USA; 4 Internal Medicine/Pulmonary and Critical Care, BronxCare Health System, Bronx, USA

**Keywords:** drug-induced thrombocytopenia, medical icu, ttp, pulmonary critical care, furosemide, hemolytic uremic syndrome, hematology, thrombotic microangiopathies, thrombotic thrombocytopenic purpura

## Abstract

Drug-induced immune thrombocytopenia (DITP) refers to drug-dependent, antibody-mediated platelet destruction. Although several drugs have been implicated as the cause of DITP, the most commonly encountered are heparin, sulfonamides, quinine, vancomycin, and beta-lactam antibiotics. However, furosemide has been rarely reported as the cause of thrombocytopenia. We present a unique case of furosemide-induced thrombotic thrombocytopenia in a 64-year-old female referred by her primary care provider for low platelets, rash, and bleeding. She was recently started on oral furosemide for diastolic heart failure two weeks before this presentation. She was admitted to the intensive care unit and was worked up for new-onset thrombocytopenia. Labs revealed anemia, thrombocytopenia, elevated lactate dehydrogenase, and low haptoglobin with normal serum creatinine. Peripheral smear showed schistocytes, low platelets, and ADAMTS13 level was 0.03. The patient was diagnosed with thrombotic thrombocytopenic purpura and treated with steroids, rituximab, and plasmapheresis, which led to rapid recovery of the platelet count to normal. Based on this case report, clinicians should consider furosemide as one of the drugs potentially causing thrombotic thrombocytopenia. Early detection and prompt management can be lifesaving.

## Introduction

Furosemide is a potent loop diuretic most commonly used in the edematous states associated with cardiac, hepatic, and renal dysfunction. It is also widely prescribed for hypertension and is used by many patients on a long-term basis. The most common drug reactions known with its use are electrolyte imbalances, which, according to Beers criteria, carry a potential warning against its use in patients over 65 years. The other common side effects are ototoxicity, interstitial nephritis, renal injury, and hypersensitivity reactions such as urticaria, angioedema, and anaphylaxis. It has also been reported that both oral and intravenous use of furosemide induces drug-dependent, platelet-reactive antibodies causing platelet destruction, thus acting as a culprit in causing drug-induced immune thrombocytopenia (DITP) [1-3].

The pathogenesis of drug-induced thrombocytopenia involves six mechanisms. The first mechanism involves a hapten-dependent antibody. The drug (hapten) links covalently to the membrane protein and induces a drug-specific immune response, as seen with penicillin, piperacillin, and cephalosporin antibiotics. The second mechanism involves a drug-dependent antibody in which the drug induces an antibody that binds to the membrane protein only in the presence of a soluble drug.

Quinine, non-steroidal anti-inflammatory drugs, and anticonvulsants use this mechanism of platelet destruction. The third mechanism involves fiban-induced thrombocytopenia. The drug reacts with membrane GPIIb/IIIa and induces conformational change recognized by naturally occurring antibodies, as seen with eptifibatide and tirofiban. The fourth mechanism involves drug-specific antibodies seen with abciximab use, in which a naturally occurring antibody or induced antibody is specific for the murine component of abciximab.

The fifth mechanism is autoantibody production, in which the drug induces an antibody that reacts with the platelets in the absence of the drug, as seen with the use of gold salts, levodopa, and procainamide. The sixth mechanism is immune-mediated platelet destruction as seen with heparin which binds to platelet factor-4 to produce a complex for which the antibody is specific. The resulting immune complex activates platelets via Fc receptors resulting in heparin-induced thrombocytopenia; however, a subset of patients experiences venous or arterial thrombosis [4]. This case discusses the use of furosemide in a patient resulting in thrombocytopenia.

## Case presentation

A 69-year-old female with a medical history of hypertension, diabetes, obesity, asthma, schizoaffective disorder, and dementia was brought to the emergency department by her son for rash and low platelets. She had been informed of her low platelets by her primary care physician, whom she saw a day before the hospital admission. According to her son, she was noted to have a bruise on her thigh two weeks earlier, which had progressively increased in size.

She also reported bleeding bilaterally from the ears and right nostril and an episode of blood in the urine a day prior. She was taken to her primary care provider and had blood work done, which showed a very low platelet count, for which they were informed to come to the emergency department. She denied any similar prior episodes of bleeding, bruising, or low platelets in the past. Family history was not significant for bleeding tendencies, easy bruising, or malignancy. Her social history was notable for being a former smoker but had stopped smoking over 20 years prior, and she denied the use of alcohol or illicit drugs.

Her previous records showed that she was admitted for diastolic heart failure with fluid overload one month back. She received intravenous (IV) furosemide in the emergency department and during her five-day stay in the hospital and was discharged on oral Lasix. Twelve days after discharge, the patient presented with a rash in the dermatology clinic. As per the son, the rash appeared post Lasix use. However, because her labs were also significant for anemia, the patient was referred to hematology. Platelets at the time of discharge were 193 k/µL.

**Figure 1:**
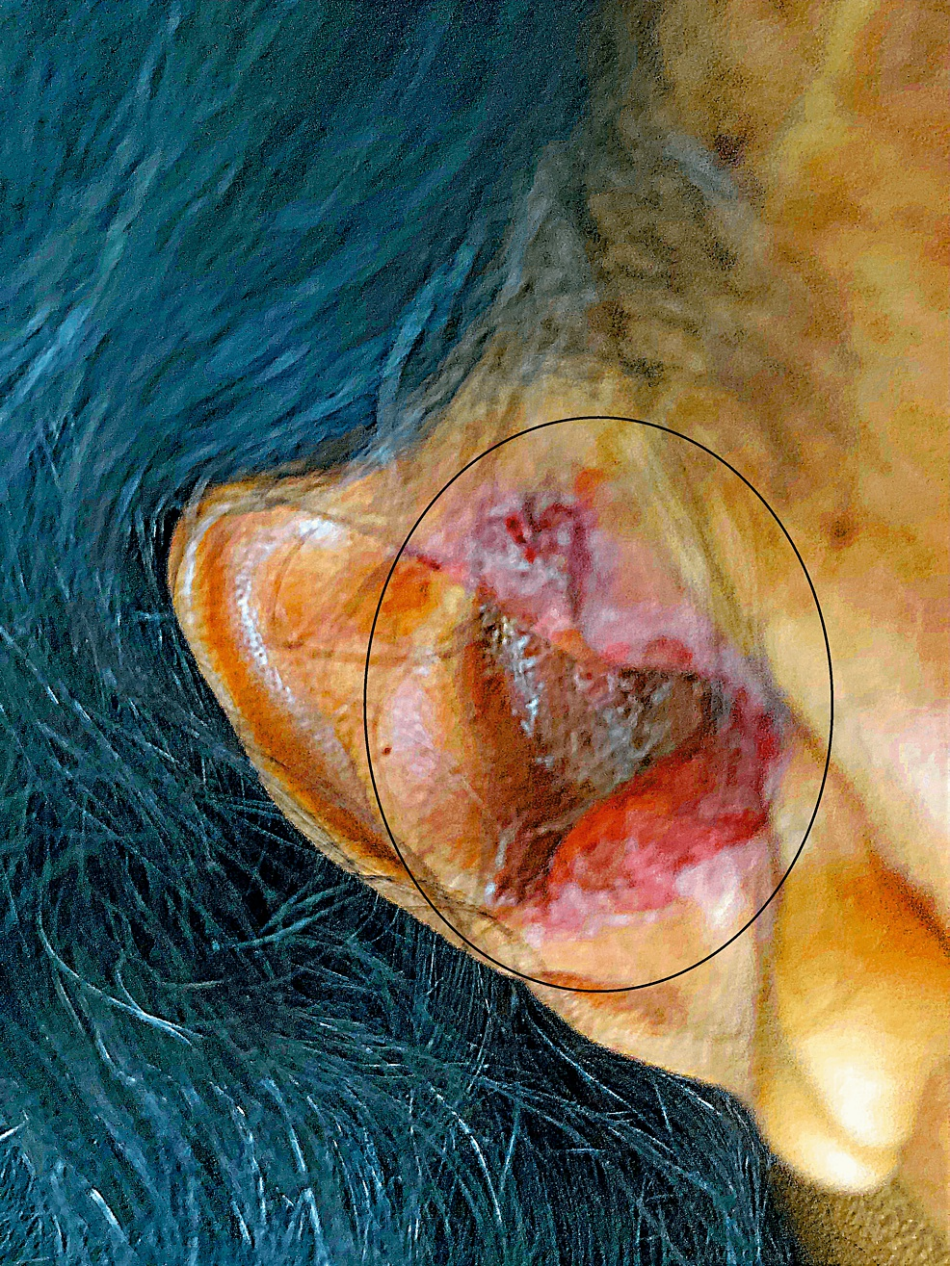
Dried crusted blood noted near the ear.

**Figure 2:**
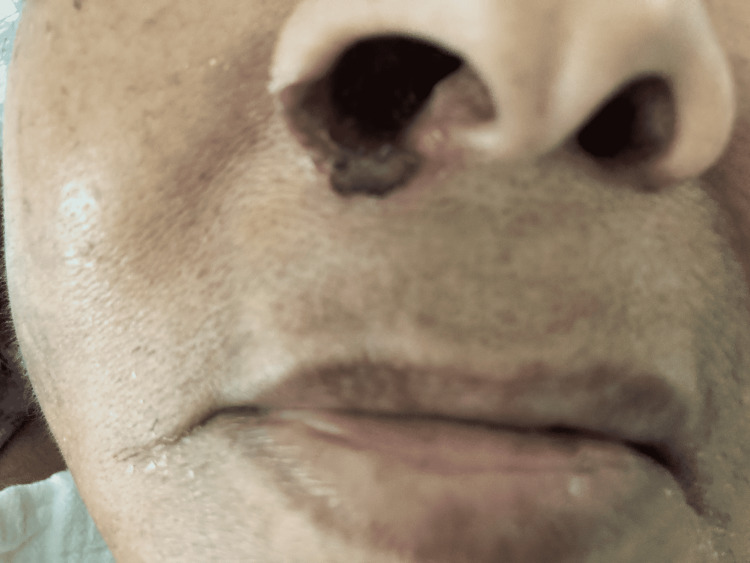
The crusted bloody lesion noted at the entrance of the right nostril.

**Figure 3:**
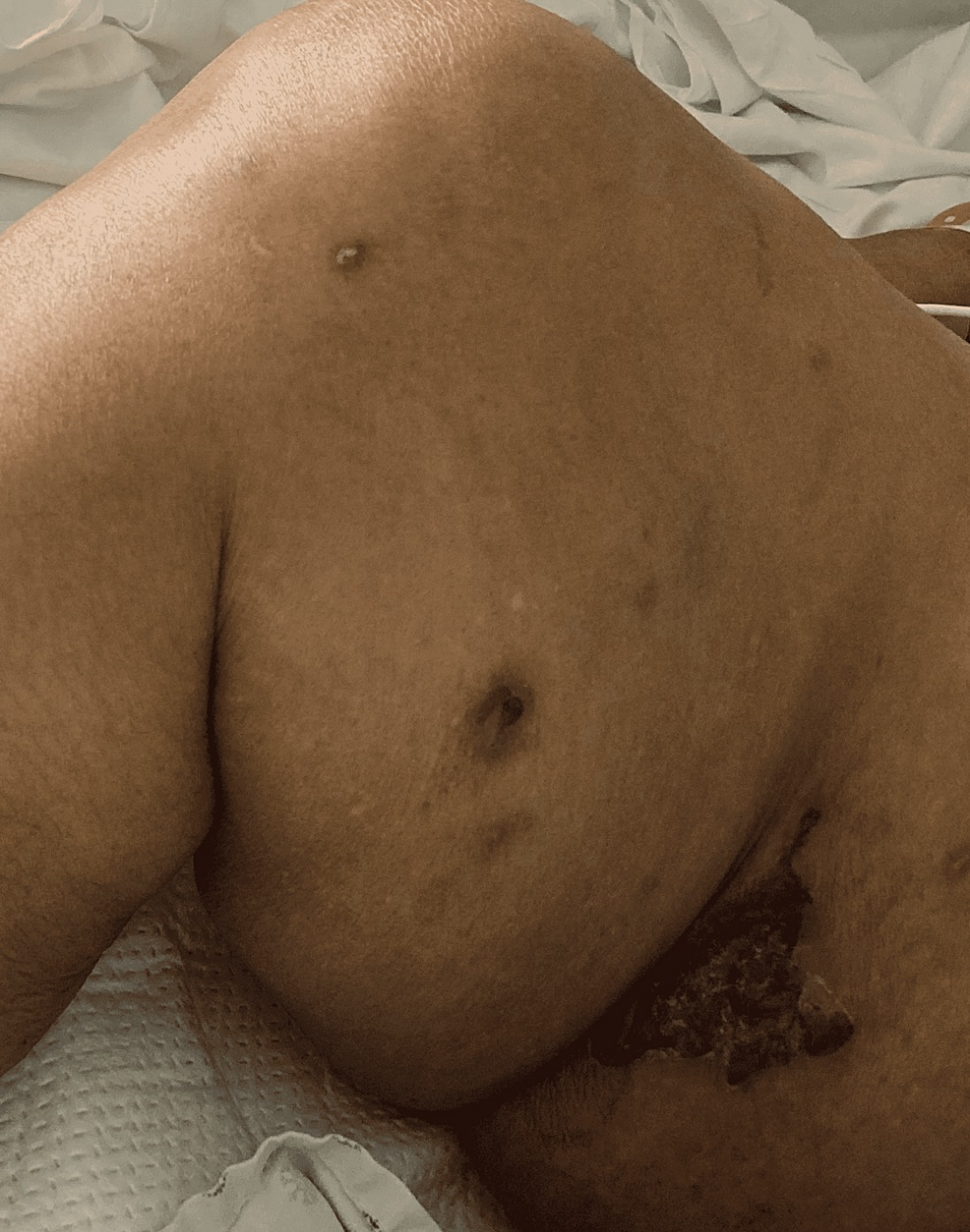
An erythematous lesion with crusted dried blood noted on the medial aspect of her right thigh.

On examination, the patient was confused and oriented only to her name and place. Dried blood was noted near the ears, and a crusted bloody lesion was noted at the entrance of the right nostril, as shown in Figure 1 and Figure 2, respectively. An erythematous lesion with crusted dried blood was noted on the medial aspect of her right thigh, as shown in Figure 3. Her vital signs were stable with a pulse rate of 86 beats per minute, blood pressure of 132/74 mmHg, a temperature of 98.1°F, and oxygen saturation of 98% on room air. The complete physical examination was unremarkable, including the lungs, cardiovascular system, and abdomen.

**Figure 4:**
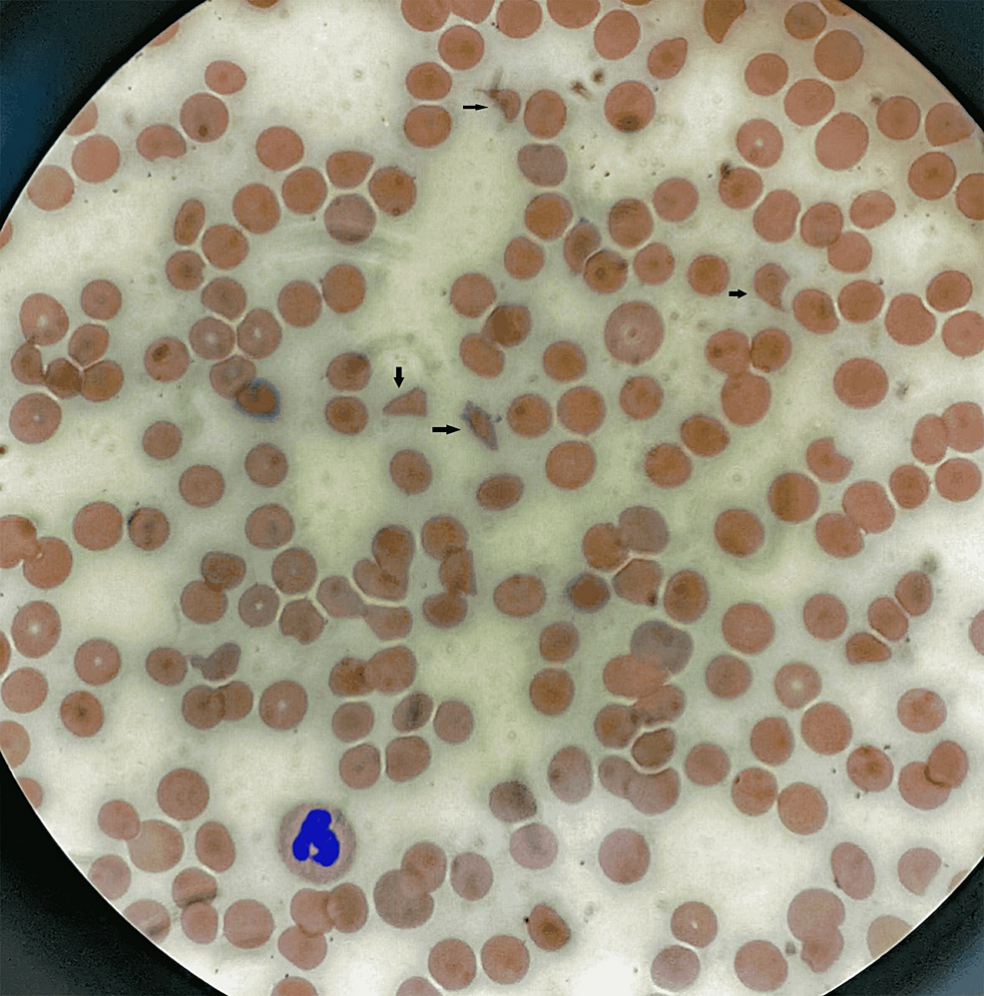
A peripheral smear revealing schistocytes (black arrows) with few platelets.

Initial laboratory investigations on admission (Table 1) were significant for anemia, thrombocytopenia, high lactate dehydrogenase (LDH), low serum haptoglobin, and an increased reticulocyte count. A peripheral smear revealed schistocytes with few platelets (Figure 4). Serum creatinine was within normal limits. The direct antibody test was negative. Chest roentgenogram and computed tomography of the head and facial bones were unremarkable.

**Table 1:** Initial laboratory values. WBC: white blood count; HgB: hemoglobin; PT: prothrombin time; INR: international normalized ratio; ADAMTS: a disintegrin and metalloproteinase with thrombospondin motifs

Complete blood count	Results	Reference range
WBC count	7.5	4.8–10.8 k/uL
HgB	9.0	12–16 g/dL
Hematocrit	27.2	42–51%
Platelets	17	150–450 k/uL
Reticulocyte count	5.1	0.5-1.5 zz
Peripheral smear	Few schistocytes, few burr cells, markedly decreased platelet cells	
General coagulation		
PT	11.1	9.9–13.3 seconds
Partial thromboplastin time	26.1	27.2–39.6 seconds
INR	0.93	0.85–1.14 seconds
General chemistry		
Serum sodium	138	135–145 mEq/L
Serum potassium	3.5	3.5–5.5 mEq/L
Serum bicarbonate	25	24–30 mEq/L
Serum creatinine	0.7	0.5–1.5 mg/dL
Lactate dehydrogenase	846	100–190 U/L
Haptoglobin	<10	30–200 mg/dL
Serum albumin	3.7	3.2–4.6 g/dl
Serum alanine aminotransferase	7	5–40 U/L
Serum aspartate transaminase	20	9–36 U/L
Serum alkaline phosphatase	98	43–160 U/L
Direct antibody testing	Negative	
ADAMTS13 activity	<0.03	0.68–1.63 IU/mL
ADAMTS inhibitor	5.5	<0.4 BEU

**Figure 5:**
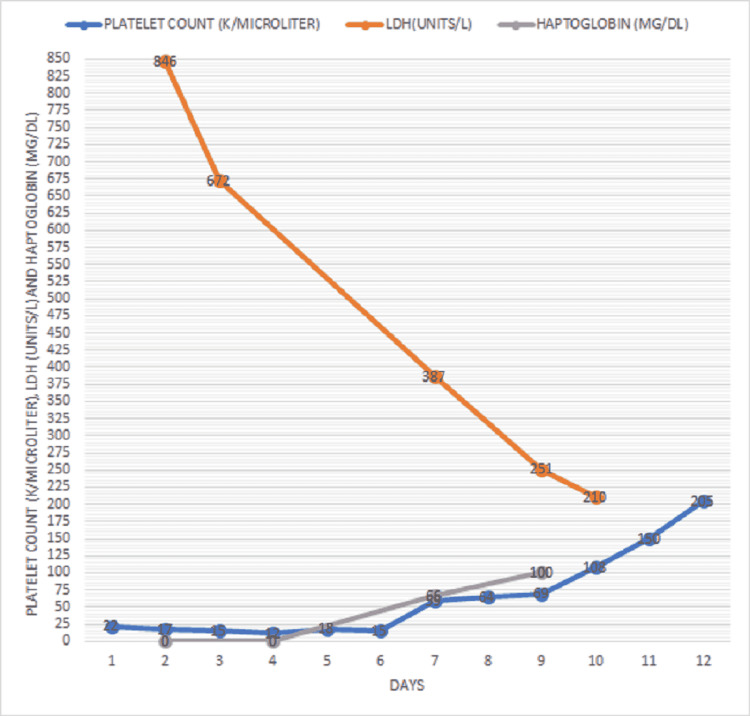
The trend of platelets, LDH, and haptoglobin over the course of treatment with plasmapheresis and steroids. LDH: lactate dehydrogenase

Our patient’s PLASMIC score was six on presentation. She had low platelets, evidence of hemolysis, low mean corpuscular volume, no evidence of active cancer, and a history of solid organ transplant with normal creatinine, which placed her in a high-risk group. The PLASMIC score was six, representing the risk of having severe ADAMTS13 (a disintegrin and metalloproteinase with thrombospondin motifs) deficiency. A clinical diagnosis of thrombotic thrombocytopenic purpura was made. According to hematology recommendations, the patient was admitted to the medical intensive care unit and started on prednisone 1 mg/kg/day. Further laboratory investigations sent were negative for human immunodeficiency virus and hepatitis B and C, and blood and urine cultures were negative. ADAMTS13 activity was reported to be <0.03 IU/mL (reference range: 0.68-1.63 IU/mL) ADAMTS inhibitor was 5.5 BEU (reference range <0.4 BEU). She underwent plasmapheresis which was continued until platelets were greater than 150,000 for two consecutive days. Her platelets improved during treatment with steroids and plasmapheresis, LDH trended down, and her haptoglobin improved, as shown in Figure 5. She was also started on rituximab and discharged with a close hematology follow-up.

## Discussion

First described by Moschcowitz in 1924, thrombotic thrombocytopenic purpura (TTP) is a rare and life-threatening condition. The annual incidence of TTP is three to eleven cases per million people. It is predominant in females, with the peak incidence in the fifth decade [5]. It is characterized by microangiopathic hemolytic anemia and severe thrombocytopenia with or without damage to the kidney and other organs [6]. TTP caused by drugs such as mitomycin, ciprofloxacin, and diclofenac has been mentioned in the literature among other drugs [7-9].

TTP is caused by severely reduced activity of the von Willebrand factor-cleaving protease ADAMTS13. This accumulates ultra-large von Willebrand factor multimers that can embolize and occlude arterioles, leading to widespread microvascular ischemia. TTP can be hereditary due to pathogenic variants in the *ADAMTS13 *gene or can be immune-mediated due to autoantibodies against ADAMTS13. DITP is one such cause and is responsible for fewer than 15% of all TTP cases [10].

Patients typically present within two weeks of exposure to the offending drug with severe thrombocytopenia (<20,000/μL) and bleeding similar to the presentation of our patient. To establish the cause-effect relationship of drug-induced thrombocytopenia in our patient, her medication list was reviewed, none of which were recently introduced to the patient’s drug list. In addition, the patient was also on heparin for deep vein thrombosis prophylaxis, which is well known to cause thrombocytopenia. However, the mechanism is drug-induced and not drug-dependent antibodies causing thrombocytopenia associated with thrombosis rather than bleeding [11].

Her presentation two weeks after its exposure falls out of the clinical time course of both heparin-induced thrombocytopenia (HIT) type 1 and HIT type 2. Patients admitted to the hospital are also given famotidine as stress ulcer prophylaxis, a rare cause of thrombocytopenia, usually seen in critically ill patients but can have numerous other reasons for thrombocytopenia [12]. Our patient was not critically sick during her prior admission and had previous multiple exposures to famotidine without effect.

ADAMTS13 levels can also be predicted by the PLASMIC score system as levels take a few days to be reported. The PLASMIC score system comprises a platelet count of <30,000/μL, evidence of hemolysis (reticulocyte count >2.5%, elevated indirect bilirubin >2 mg/dL, undetectable to low haptoglobin levels), creatinine <2 mg/dL, mean corpuscular volume <90 fL, and international normalized ratio <1.5, with no active cancer or organ/stem cell transplant [13,14]. Our patient’s PLASMIC score was six, representing the risk of having severe ADAMTS13 deficiency.

The new drug introduced to her was furosemide; she received both IV and oral formulations and presented with rash, thrombocytopenia, and bleeding days after its exposure, making it the most likely culprit causing DITP. Re-exposure to furosemide during this admission led to a fall in platelets from 21 k/μL to 15 k/μL. It was promptly recognized and discontinued.

Management involves discontinuing the drug, hospitalization depending on the severity of the symptoms, and initiation of therapeutic plasma exchange based on the clinical diagnosis and later confirmed diagnosis of severely deficient ADAMTS13 activity. In addition, monoclonal antibodies such as rituximab and caplacizumab should be administered after therapeutic plasma exchange to prevent a recurrence. Glucocorticoids also play an essential role as they aid in reducing the production of ADAMTS13 inhibitors. Early recognition and establishing a diagnosis of DITP are essential to ensure prompt discontinuation of the offending agent, early treatment, and avoiding re-exposure to the drug.

## Conclusions

Furosemide is one of the most extensively used drugs in patients with volume overload. Patients receiving furosemide and presenting with thrombocytopenia should be investigated appropriately for immune-mediated TTP. Early detection and prompt management can be lifesaving.
